# Why Are We Frequently Ordering Urinalyses in Patients without Symptoms of Urinary Tract Infections in the Emergency Department?

**DOI:** 10.3390/ijerph191710757

**Published:** 2022-08-29

**Authors:** Tessa M. Z. X. K. van Horrik, Bart J. Laan, Allard B. Huizinga, Gercora Hoitinga, Walter P. Poortvliet, Suzanne E. Geerlings

**Affiliations:** 1 Department of Internal Medicine, Division of Infectious Diseases, Amsterdam UMC, University of Amsterdam, Room D3-226 Meibergdreef 9, 1105 AZ Amsterdam, The Netherlands; 2 Department of Emergency Medicine, Amsterdam UMC, University of Amsterdam, 1105 AZ Amsterdam, The Netherlands; 3 Department of Emergency Medicine, Meander MC, 3813 TZ Amersfoort, The Netherlands

**Keywords:** urinalysis, emergency services, antimicrobial stewardship, quality improvement, urinary tract infections

## Abstract

(1) Background: In the emergency department (ED), ordering urine tests in patients without symptoms of a urinary tract infection can lead to inappropriate antimicrobial treatment. We aimed to identify factors contributing to the unnecessary ordering of urinalyses in the ED. (2) Methods: An online survey study among nurses and physicians working in the EDs of five hospitals in the Netherlands was conducted. (3) Results: The overall response rate was 26% (221/850; 85 nurses and 136 physicians). The vast majority of the respondents reported knowing when to order urine tests (197/221; 90%). Almost two-thirds of the respondents (145/221; 66%) agreed that they ordered urinalyses because it is rapid and non-invasive to patients. Most nurses (66/86; 78%) said they informed the doctor if they thought the urine test would not contribute to the patient’s diagnosis, but only one-third of the physicians agreed with this statement (44/136; 32%). Most respondents (160/221; 72%) thought guidelines or protocols about urinalyses in the ED would be functional. (4) Conclusions: These results suggest urinalyses were frequently ordered in the ED to achieve a fast work process. Nurses and physicians could improve their communication about the indications for urine tests. Developing diagnostic guidelines for urine testing may be convenient.

## 1. Introduction

Urine tests, such as urinalyses and urine cultures, are frequently ordered in the emergency department (ED) [1,2]. A urinalysis is ordered for a variety of indications, for instance, the diagnosis or cause of urinary tract infections (UTIs), kidney failure, or hyperglycemic crisis [3]. Furthermore, urinalyses are considered cheap and non-invasive to patients and therefore easy to order in patients in the ED. However, positive urinalysis results for nitrite or leukocytes can lead to inappropriate diagnosis and antimicrobial treatment of UTIs in asymptomatic patients [4,5,6]. Patients without symptoms of a UTI but with positive urine test results are defined as having asymptomatic bacteriuria (ASB). Antimicrobial treatment of ASB is considered unnecessary in most patients by the Infectious Diseases Society of America guidelines for ASB and national clinical practice guidelines for UTIs in the Netherlands [5,7,8].

In 2018, an implementation guide was provided to reduce overtreatment of ASB through the use of antimicrobial stewardship interventions [9]. A qualitative study that was conducted among 21 physicians of a tertiary care hospital in Switzerland in 2016 identified multiple reasons that contribute to the overtreatment of ASB [10]. These reasons included broad screening urinalysis, anxiousness, insecurity, overcautiousness of physicians, and time pressure. Further, in 2018, a focus group study was performed in a veterans hospital in the United States of America with five nurses in the ED and six nurses in the intensive care unit [11]. This study identified that patient factors and a lack of communication between nurses and physicians about urinalysis orders affect concordance with recommended urine ordering and collection practices [11].

Urinalyses and urine cultures are also frequently ordered in EDs in the Netherlands [12]. In order to reduce overtreatment of ASB, a quality improvement project was initiated in the EDs of five hospitals in the Netherlands [13]. The aim of the current study was to identify factors contributing to inappropriate urine testing in EDs as part of the problem analysis of this quality improvement project. The results of this study will be used to develop additional strategies to improve diagnostic stewardship to reduce overtreatment of ASB.

## 2. Materials and Methods

### 2.1. Study Design and Setting

This study was part of the cluster-randomized trial Reduce Overtreatment of Asymptomatic Bacteriuria (ROAB) [13]. We performed an online survey study in the EDs of five hospitals (one university hospital and four teaching hospitals) that participated in this study from October to December 2021. In The Netherlands, patients in the ED are not only seen by emergency physicians but also by residents or physicians of other specialties. Therefore, we invited all ED nurses, emergency physicians, and physicians working in the ED (i.e., internal medicine, surgery, geriatrics, and neurology) to participate in our survey.

We distributed the survey by e-mail from the administration offices, the medical specialists involved in the ROAB study, and the department’s newsletters. An e-mail reminder was sent two weeks after the first invitation. In total, 850 nurses and physicians received the invitation to the survey. Participation in this survey was anonymous, could be quit anytime, and there were no incentives.

### 2.2. Survey and Data Collection

Since there were no validated surveys regarding this topic available, we conducted an open electronic survey for nurses and physicians in the Dutch language. The surveys were developed in close collaboration with the chair (S.E.G.) of the Dutch Working Party on Antibiotic Policy (Dutch acronym: SWAB) for Urinary Tract Guidelines Committee 2020 [8,14].

The surveys consisted of 16 and 15 statements and three patient case questions for nurses and physicians, respectively, and took five to ten minutes to complete. We used Microsoft Forms (Microsoft 2021^®^) to distribute the surveys and collect the data. We did not use adaptive questioning or randomization of the items in the survey. Participants were able to review and change their answers during the filling in of the survey. Further, no personal data, such as unique visitor rate or registration, were tracked, as this was an open and completely anonymous survey.

The content of both surveys was divided into the following parts: baseline characteristics, self-assessment, behavior, guidelines, and patient cases. We based the statements on the results of previous studies that identified different factors underlying the request for urine diagnostics and antibiotic treatment of ASB [10,11,15,16,17]. For all statements, we used a 5-point Likert scale (1: ‘strongly disagree’ to 5: ‘strongly agree’). In the patient cases, we asked whether the participant would order a urinalysis for this patient, and we considered the correct answers based on the IDSA guideline for the management of ASB [5]. We did not formally validate the surveys, but both surveys were proofread by colleagues.

### 2.3. Data Analysis

For the data analysis, we included all surveys that were fully completed. We considered the three general practitioners in training who were working in the ED as emergency residents in the analysis of the data. Further, one clinical nurse specialist in training and one physician assistant in training, whose work tasks were similar to those of a physician from their department, were considered physicians in the data analysis. Physicians were residents or medical specialists. The response rates were calculated by dividing the number of included surveys by the number of recipients of the survey as reported by the administration offices. The categorical data are presented as frequencies and percentages. We used descriptive statistics to assess the data. No sample size was calculated for this study since this study was conducted as a problem analysis. We used IBM SPSS Statistics for Windows version 26.0 (IBM Corp., Armonk, NY, USA) for the statistical analyses.

## 3. Results

### 3.1. Participants and Response Rates

In total, 850 nurses and physicians received the survey, and we received 221 responses from 85 nurses and 136 physicians. All surveys were fully completed. The overall response rate was, therefore, 26% (221/850). The number of responses from the five hospitals varied between 31 and 53 (14–24% of the total number of responses). Almost one-third of the ED nurses had >25 years of work experience, while almost two-thirds of the physicians had 1–5 years of work experience. Half of the physicians worked in a non-surgical department (70/136; 51%). All participant characteristics are shown in Table 1.

### 3.2. Healthcare Workers’ Self-Reported Knowledge of Urine Testing

Almost all nurses (81/85) and physicians (116/136) reported that they knew when they must order a urine test (Table 2). None of the medical specialists and emergency physicians reported they did not know when to order urine tests in contrast to five residents. The majority of the respondents (55/85 nurses and 101/136 physicians) reported feeling confident about their interpretation of a urinalysis result. Further, slightly more than half of the nurses (44/85) compared to 85% (116/136) of the physicians were familiar with ASB. Approximately one-third (26/85) of the nurses thought ASB is generally harmful to patients.

Regarding the patient cases, half of all respondents (51/85 nurses and 66/136 physicians) would order urinalysis in an elderly female patient with back pain who did not have any symptoms of confusion (Table 3). Of these 51 nurses, 5 were in training (36% of all nurses in training), and 43 were not in training (61% of all nurses not in training). Regarding these 66 physicians, 55 were residents (49% of all residents), 8 were emergency physicians (67% of all emergency physicians), and 3 were medical specialists (27% of all medical specialists). Concerning the second patient case, 21/113 (27%) residents, 1/11 (9%) medical specialists, and 2/12 (17%) emergency physicians would obtain urinalysis in the absence of fever or urogenital symptoms.

### 3.3. Work Process Factors Related to Inappropriate Urine Testing

Almost two-thirds of the nurses (54/85) reported that they thought routine ordering of urine tests should be reduced in the ED (Table 4). On the other hand, a minority of the physicians (49/146) agreed with this statement. Further, most of all respondents (66/85 nurses and 94/136 physicians) reported that they thought guidelines or protocols about urine diagnostics would be useful in the ED. However, only a minority of all respondents (8/85 nurses and 22/136 physicians) reported the actual use of a guideline or protocol for UTI diagnostics.

Most of the nurses (64/85) and physicians (78/136) reported that nurses ordered urinalyses independently. Further, the vast majority of the respondents (69/85 nurses and 83/113 residents, 11/12 emergency physicians, and 5/11 medical specialists) reported that nurses ordered urinalyses in the ED before the patient was examined by a physician due to the fast workflow in the ED. In addition, many nurses (46/85) and physicians (100/136) agreed that they ordered urinalyses because they considered this test rapid and non-invasive for patients. The percentages of residents (89/113; 79%) and emergency physicians (9/12; 75%) that agreed with these statements were higher than the percentage of medical specialists (2/11; 9%).

### 3.4. Communication Factors Related to Reducing Inappropriate Urine Testing

The majority of the nurses (66/85) responded that they informed physicians if they thought that a urine test would not contribute to a patient’s diagnosis (Table 5). Further, the majority of the nurses (65/85) and physicians (84/136) reported that nurses would not obtain urine cultures from patients without consulting the physician. Additionally, over half of the nurses (45/85) reported that physicians took their judgment into consideration before ordering urine tests. However, 59/113 (53%) residents, 4/12 (33%) emergency physicians, and 7/11 (64%) medical specialists reported that nurses did not inform them about their judgment in a follow-up question. In addition, almost half of the nurses (38/85) reported that they were criticized by physicians if they did not follow their requests.

## 4. Discussion

In this study, we aimed to identify factors related to inappropriate urine testing in five EDs in the Netherlands. Nurses and physicians felt confident about their knowledge about ASB and urine testing but did not give the correct answers to all patient cases. Most nurses thought that a protocol for urine testing could be helpful in the ED. Barriers to reducing inappropriate urine testing could be the consideration of the urinalysis as a rapid and non-invasive diagnostic tool, the fast workflow in the ED, and different views on communication between nurses and physicians regarding ordering urine tests. Specific knowledge about ASB could be of added value in reducing inappropriate ordering of urine tests.

We found differences in perceived knowledge of and views on urine testing from nurses and physicians that could be due to the different education and work tasks [18]. Regarding the statements on routine urine testing in the ED, it is interesting that more nurses than physicians reported that this should be reduced. Nurses possibly felt as though they ordered more routine urine tests than physicians due to following the local operational protocols that were mainly developed by physicians [17]. In addition, the results of a recently performed study showed that urinalyses were more likely to be ordered by mid-level providers (e.g., nurse practitioners or physician assistants) than physicians [2]. Further, we hypothesized that fewer physicians than nurses agreed that routine urine tests should be reduced because this would obstruct the fast work process and prolong a patient’s length of stay in a crowded ED [19]. Next to these reasons, nurses probably ordered urine tests without consulting the doctor to avoid delaying the work process and because they anticipated previously given orders or established diagnostic routines of certain medical specialties. For example, when the internal medicine specialist is consulted, the ED nurse already knows that this physician would want a urine test. Therefore, the ED nurse would order urine tests without consulting this physician to not obstruct the diagnostic procedures in the ED.

Interestingly, only a few physicians reported being afraid of missing out on diagnosing a UTI, suggesting this factor would not contribute to the overuse of urinalysis. However, it might be possible that physicians ordered urine tests for almost all of their patients in the ED, causing them not to be afraid of missing out on diagnosing UTIs.

The work process and communication factors we identified in this study were similar to those of previously performed barrier and facilitator analyses of reducing inappropriate urine testing [10,16]. Another important factor could be the crowding in the ED, which could cause ED nurses and physicians to order urine tests, aiming to reduce a patient’s length of stay in the ED to a minimum. In addition, the features of the urinalysis itself, namely cheap, non-invasive, and rapid, also seemed to promote its overuse in the crowded ED [3,20]. Further, the answers to the patient cases revealed that approximately half of the physicians and nurses would order urinalysis in an elderly patient with back pain but without signs of confusion or a patient with a urinary catheter who had cloudy urine. These patient factors were also identified in previous studies that identified confusion and change in urine aspects as risk factors for inappropriate urine testing and overtreatment of ASB [6,21]. Our results indicate the importance of educating healthcare workers about appropriate indications for urine testing.

Clear guidelines and protocols about urine testing and interpreting urine test results were lacking in EDs in the Netherlands as most of these generally focused on antimicrobial treatment [7,8]. In order to reduce inappropriate urine testing, we recommend addressing appropriate indications for urine testing in diagnostic guidelines in addition to the therapeutic guidelines and protocols. Further, nurses should be included in diagnostic and antimicrobial stewardship interventions since they ordered most of the urine tests in the initial assessment of the patients admitted to the ED [18,22]. Additionally, enhancing knowledge of ASB among healthcare workers in the ED might contribute to reducing inappropriate urine testing since almost one-third of the nurses considered ASB harmful to patients, which could influence their ordering of urine tests [23]. In order to promote appropriate ordering of urine tests, it might be convenient to build in reminders, alerts, or order sets with automatic cancellations of urine tests based on certain parameters in the patient record, but it is important that this does not affect the safety in terms of clinical care for patients. In addition to clinically relevant reasons to reduce the inappropriate ordering of urine tests in the absence of UTIs, the financial aspect could be substantial. In a previous study performed in seven hospitals in the Netherlands, we found that canceling urine testing orders after a negative dipstick would have saved almost EUR 19,500 during the study period of that trial in seven hospitals [12].

A strength of this study was that we included participants from EDs of five hospitals in the Netherlands, making the results more generalizable. We believe this study reached an adequate response rate of 26%, even though we invited our participants during a peak in the COVID-19 pandemic. Furthermore, the majority of the included physicians were residents, indicating that our results reflect the experiences of healthcare workers working in the ED. However, this study also had some limitations. Firstly, we could not use a validated survey on this topic, but we based the statements of our survey on previously identified targets for diagnostic stewardship and urine testing. Secondly, most of the participating physicians in our survey worked in a non-surgical department, which could lead to a selection bias. This was probably due to the fact that this study was initiated by the internal medicine departments and the physicians, who needed to forward the invitations, were internal medicine specialists. However, urine tests are generally most frequently ordered by the physicians of the internal medicine department. Therefore, the possible selection bias is probably negligible. Thirdly, a common problem in this type of study is the social desirability bias, which is visible in the results of the respondents that scored high on the self-assessment statements (e.g., knowledge and confidence in interpreting urine test results), yet scored moderately on the patient cases. Further, our study mainly focused on the overtreatment of ASB. However, urinalysis can be used for a variety of clinical indications in the ED (e.g., proteinuria, glycosuria), especially because it is a cheap and non-invasive test. Lastly, the results of this study did not provide extensive information about the patient factors and patient-related indications for urine testing. Regarding these patient factors, we are currently analyzing the results of the ROAB study, in which we will assess the actual indications for urine testing in that study [13]. We will use these results to develop additional diagnostic stewardship strategies to reduce overtreatment of ASB.

## 5. Conclusions

The urinalysis is frequently used because of its favorable features to avoid prolonged waiting times in the diagnostic procedures and thus minimize the length of stay in the ED. In order to prevent asymptomatic patients with a positive urine test result from being inappropriately treated with antimicrobials, guidance about indications to perform a urinalysis for nurses and for the interpretation of urine tests for physicians could be convenient.

## Figures and Tables

**Table 1:** Participant characteristics.

Characteristic	Nurses (*n* = 85)	Physicians (*n* = 136)
Female	69 (81%)	85 (63%)
Male	15 (18%)	50 (37%)
I don’t want to say	1 (1%)	1 (1%)
**Work place**		
Hospital 1	21 (25%)	32 (24%)
Hospital 2	28 (33%)	21 (15%)
Hospital 3	13 (15%)	28 (21%)
Hospital 4	18 (21%)	29 (21%)
Hospital 5	5 (6%)	26 (19%)
**Work experience in years**		
1–5	8 (9%)	89 (65%)
5–10	14 (16%)	24 (18%)
10–15	13 (15%)	12 (9%)
15–20	10 (12%)	3 (2%)
20–25	13 (15%)	6 (4%)
>25	27 (32%)	2 (2%)
**Profession**		
ED nurse *	71 (84%)	n.a.
ED nurse in training	14 (16%)	n.a.
Non-surgical resident **	n.a.	70 (52%)
Surgical resident	n.a.	20 (15%)
Emergency physician	n.a.	12 (9%)
Neurology resident	n.a.	11 (8%)
Medical specialist ***	n.a.	11 (8%)
Emergency resident	n.a.	10 (7%)
Other department	n.a.	2 (1%)

ED nurse: emergency department nurse; percentages may not add up to 100% due to rounding; n.a. = not applicable; * including 1 pediatric ED nurse; ** internal medicine and other non-surgical specialties (e.g., pulmonology, gastroenterology); *** 1 surgeon and 10 non-surgical medical specialist (e.g., internal medicine specialist).

**Table 2:** Healthcare workers’ knowledge about urine testing.

Statement	Nurses (*n* = 85)	Fully Disagree	Disagree	Neutral	Agree	Fully Agree
	Physicians (*n* = 136)					
I know when I must order a urine dipstick, microscopic analysis, or urine culture.	Nurses	0	0	4 (5%)	61 (72%)	20 (24%)
	Physicians	0	5 (4%)	15 (11%)	87 (64%)	29 (21%)
I feel confident about my interpretation of a urinalysis result.	Nurses	0	2 (2%)	28 (33%)	44 (52%)	11 (13%)
	Physicians	0	10 (7%)	25 (18%)	83 (61%)	18 (13%)
I think a positive urinalysis result always indicates a urinary tract infection.	Nurses	0	23 (27%)	23 (27%)	38 (45)	1 (1%)
	Physicians	10 (7%)	83 (61%)	22 (16%)	19 (14%)	2 (2%)
I know what asymptomatic bacteriuria is.	Nurses	2 (2%)	18 (21%)	19 (22%)	43 (51%)	3 (4%)
	Physicians	1 (1%)	6 (4%)	13 (10%)	84 (62%)	32 (24%)
In general, asymptomatic bacteriuria is not harmful to patients.	Nurses	1 (1%)	25 (29%)	36 (42%)	23 (27%)	0
	Physicians	0	8 (6%)	14 (10%)	100 (74%)	14 (10%)
Generally, I am afraid of missing out on diagnosing a urinary tract infection.	Nurses	n.a.	n.a.	n.a.	n.a.	n.a.
	Physicians	18 (13%)	88 (65%)	17 (13%)	13 (10%)	0

Data are *n* (%); percentages may not add up to 100% due to rounding; n.a. = not applicable.

**Table 3:** Number of correct answers to patient cases.

Case	Correct Answer *	Nurses (*n* = 85)	Physicians (*n* = 136)
1. 83-year-old female with back pain, without signs of confusion.	No	37 (44%)	70 (52%)
2. 75-year-old male with urinary catheter and cloudy urine, but without any abdominal pain.	No	44 (52%)	102 (75%)
3. 34-year-old pregnant female with abdominal pain.	Yes	77 (91%)	130 (96%)

For each case, the question was: For whom of the following patients, who do not have a fever or any urogenital symptoms, would you order urinalysis? Symptoms and comorbidities that are not described in the case are absent. * According to Infectious Diseases Society of America Guideline on asymptomatic bacteriuria 2019 [5].

**Table 4:** Work process factors related to inappropriate urine testing.

Statement	Nurses (*n* = 85)	Fully Disagree	Disagree	Neutral	Agree	Fully Agree
	Physicians (*n* = 136)					
I order urinalyses because I believe it is a rapid and non-invasive diagnostic tool.	Nurses	4 (5%)	24 (28%)	11 (13%)	44 (52%)	2 (2%)
	Physicians	0	16 (12%)	20 (15%)	83 (61%)	17 (13%)
I order urinalysis in patients before the doctor has seen or examined them because of the fast work process in the ED.	Nurses	1 (1%)	9 (11%)	6 (7%)	52 (61%)	17 (20%)
	Physicians	3 (2%)	20 (15%)	14 (10%)	73 (54%)	26 (19%)
Fewer routinely ordering of urinalyses is needed in the ED.	Nurses	0	11 (13%)	20 (24%)	37 (44%)	17 (20%)
	Physicians	1 (1%)	4 (3%)	37 (27%)	79 (58%)	15 (11%)
I order urinalyses independently, without consulting the doctor.	Nurses	0	6 (7%)	15 (18%)	56 (66%)	8 (9%)
	Physicians	n.a.	n.a.	n.a.	n.a.	n.a.
I think guidelines or protocols about urinalysis in the ED would be convenient.	Nurses	0	2 (2%)	17 (20%)	54 (64%)	12 (14%)
	Physicians	1 (1%)	4 (3%)	37 (27%)	79 (58%)	15 (11%)

Data are *n* (%); percentages may not add up to 100% due to rounding; ED = emergency department; n.a. = not applicable.

**Table 5:** Communication factors related to reducing inappropriate urine testing.

Statement	Nurses (*n* = 85)	Fully Disagree	Disagree	Neutral	Agree	Fully Agree
	Physicians (*n* = 136)					
Nurses order urine cultures without consulting the doctor, even if the urinalysis result is negative.	Nurses	17 (20%)	48 (57%)	11 (13%)	9 (11%)	0
	Physicians	21 (15%)	63 (46%)	26 (19%)	23 (17%)	3 (2%)
Nurses will tell the doctor if they do not find a urinalysis convenient in diagnosing a patient.	Nurses	0	7 (8%)	12 (14%)	59 (69%)	7 (8%)
	Physicians	14 (10%)	56 (41%)	22 (16)	41 (30%)	3 (2%)
Doctors take nurses’ judgment into consideration when ordering urinalyses.	Nurses	2 (2%)	8 (9%)	30 (35%)	43 (51%)	2 (2%)
	Physicians	n.a.	n.a.	n.a.	n.a.	n.a.
I get criticism from doctors if I do not carry out their assignment(s).	Nurses	3 (4%)	14 (17%)	30 (35%)	34 (40%)	4 (5%)
	Physicians	n.a.	n.a.	n.a.	n.a.	n.a.

Data are *n* (%); percentages may not add up to 100% due to rounding; n.a. = not applicable.

## Data Availability

The datasets are available from the corresponding author on reasonable request.
